# BioDataome: a collection of uniformly preprocessed and automatically annotated datasets for data-driven biology

**DOI:** 10.1093/database/bay011

**Published:** 2018-03-02

**Authors:** Kleanthi Lakiotaki, Nikolaos Vorniotakis, Michail Tsagris, Georgios Georgakopoulos, Ioannis Tsamardinos

**Affiliations:** 1 Computer Science Department, University of Crete, Voutes Campus, 70013 Heraklion, Crete, Greece; 2 Gnosis Data Analysis PC, Palaiokapa 64, 71305 Heraklion, Crete, Greece

## Abstract

Biotechnology revolution generates a plethora of omics data with an exponential growth pace. Therefore, biological data mining demands automatic, ‘high quality’ curation efforts to organize biomedical knowledge into online databases. BioDataome is a database of uniformly preprocessed and disease-annotated omics data with the aim to promote and accelerate the reuse of public data. We followed the same preprocessing pipeline for each biological mart (microarray gene expression, RNA-Seq gene expression and DNA methylation) to produce ready for downstream analysis datasets and automatically annotated them with disease-ontology terms. We also designate datasets that share common samples and automatically discover control samples in case-control studies. Currently, BioDataome includes ∼5600 datasets, ∼260 000 samples spanning ∼500 diseases and can be easily used in large-scale massive experiments and meta-analysis. All datasets are publicly available for querying and downloading via BioDataome web application. We demonstrate BioDataome’s utility by presenting exploratory data analysis examples. We have also developed BioDataome R package found in: https://github.com/mensxmachina/BioDataome/.

Database URL: http://dataome.mensxmachina.org/

## Introduction

In the face of unprecedented amounts of genomics data generated by the molecular biology revolution, researchers have developed a massive number of databases aiming to deal with gigantic volumes of biological data. Retrieving, preprocessing and curating biological data are nontrivial, time-consuming and error-prone. Instead, pooling and sharing such data significantly accelerate scientific progress, but questions remain about how these data will impact research practice, from wet-lab biologists to bioinformaticians. In a 2012 review (1), authors point out the power of reusing public gene expression data, spanned mainly in three directions: (i) the study of a biological question, (ii) the development and evaluation of a new method and (iii) the integration, annotation and analysis of primary data in order to build a new, value-added data resource.

Biological databases are being developed with a different purpose, structure and data; however, it remains a crucial fact that biological sense can only fully be made from genomics data when accurate and adequate contextual information is available. This information is essential for data to be discoverable by the user and to lead to deep interpretation. Besides the importance of standardized annotation on discoverability and downstream use of genomics datasets, sufficient contextual annotation is frequently lacking in public biomedical data sets (2, 3). Hooper *et al.* (3) also emphasize its significant costs and the need for a submitter-driven annotation system as a sustainable curation solution. 

Regardless of the use they serve, biological databases developed nowadays should follow BioDBcore’s guidelines and standards (http://biocuration.org/community/standards-biodbcore/), the findable, accessible, interoperable and reusable data principles and any related standards defined by the functional genomics data society, like the minimum information about a microarray experiment or minimum information about a high-throughput nucleotide SEQuencing experiment for microarray or high-throughput sequencing gene expression data, such as Illumina, formerly next-generation Sequencing, respectively. Biological database developers must also ensure that their database is accurately listed in catalogs like biosharing.org (4).

As several biological data repositories are being developed, the need for tools and services to find, access and use those repositories becomes apparent. DataMed, e.g. is an ongoing project for the development of a biomedical data search engine to discover data sets across multiple data repositories by keyword search (https://datamed.org/). It is supported by the National Institutes of Health (NIH) Big Data to Knowledge. Repositive, an online platform that indexes genomic data also offers a single place to search for and access a range of human genomic data.

Besides tools to search and retrieve biological data, data curation projects are making great progress mining biomedical literature to extract and aggregate decades worth of research findings. inSilicoDB (5), an open data management and access platform, allows programmatic download of gene expression data curated by maintainers or contributors from the community, however users may only retrieve one experiment at a time. In GENEVESTIGATOR (6), authors claim to offer quality controlled, normalized and carefully manually annotated by experts, microarray and RNA-Seq expression data from several organisms, however GENEVESTIGATOR is a commercial product offering a free limited use to academics. Automated repository acquisition (ARepA) (7) retrieves heterogeneous data from multiple public repositories in a uniform environment and format; however retrieved data are normalized for between-dataset comparison and thus, it is restricted to a per pipeline normalization. Moreover, ARepA is a command-line tool supported by Linux and MacOS only. Microarray meta-analysis database (M2DB) (8) is a human curated microarray database with manually curated sample annotations developed to promote meta-analysis. However, M2DB contains data only from two human microarrays and although it applies three of the most widely used preprocessing algorithms [MAS5, RMA (Robust Multiarray Average) and GCRMA (GC Robust Multiarray Average)], these are array-dependent algorithms and cannot preprocess samples separately. Single array preprocessing algorithms, like frozen Robust Multiarray Average (fRMA) (9) or Single-channel array normalization (SCAN) (10), have several advantages over multi-array alternatives (11).

Rapid accumulation of vast amounts of microarray data in public databases like gene expression omnibus (GEO) (12) and ArrayExpress (13) over the past few years has now made it possible to retrieve, integrate and compare microarray results from many datasets. Tools that focus on differential gene expression analysis and integrate or compare data based on gene expression profiles have been developed. GeoDiver (14) is an online web application for performing differential gene expression analysis and generally applicable gene-set enrichment analysis on gene expression datasets from the publicly available GEO. Gemma (15), is a resource for the reuse, sharing and meta-analysis of expression profiling data. ExpressionBlast (16) compares gene expression data with thousands of other studies in order to find experiments with similar profiles. Bgee (17) retrieves and compares gene expression patterns in multiple animal species produced from multiple data types. Harmonizome (18) integrates data about genes and proteins from many online resources into ∼72 million functional associations between genes/proteins and their attributes.

As the development of structured, and thus suitable for integration data increases, along with the development of tools that integrate data from various databases to advance biomolecular knowledge, the need for uniformly preprocessed datasets, automatically annotated based on established ontologies and ready for downstream analysis will grow in parallel. Some of the current approaches mentioned earlier, often need manual curation and data annotation thus is limited to certain samples. They also don’t ensure a uniform preprocessing, may be commercial or not updated.

Even though lack of standardization in biological databases has been brought to the attention of the research community long time ago and significant progress has been done towards that direction, it remains an issue. Inspired by the need to reuse public omics data (1) and help uncover insights buried in this vast amount of data, and driven by the lack of standardization in biological databases, we developed BioDataome, a web-based tool, which addresses those challenges. In BioDataome, we have downloaded, preprocessed and annotated several thousands of omics data. All raw data have been retrieved from GEO and recount (19). Currently, BioDataome holds ∼5600 datasets from five different microarray gene expression technologies (GPL570, GPL96, GPL6244 and GPL1261), the GPL13534 Human Methylation BeadChip from GEO and the GPL11154 high-throughput sequencing technology from recount. Most of the samples are Homo Sapiens (243 563 samples) and the rest are Mus Musculus (20 723 samples). BioDataome is updated bi-weekly with ∼500 samples and summary statistics can be found on its home page (http://dataome.mensxmachina.org/). In BioDataome, users can download uniformly preprocessed omics data and perform complex queries to design their analysis and develop tools that integrate biomedical knowledge. We have also developed BioDataome R package that contains all the functions used to download, preprocess and annotate all the datasets and BioDataome vignettes, which provides a task-oriented description of BioDataome package, examples of user interaction with BioDataome and some examples of analyzing BioDataome datasets.

## Datasets and resources

### Data resources

#### GEO

GEO (12) is a database supported by the National Center for Biotechnology Information (NCBI) at the National Library of Medicine (NLM), which accepts raw and processed data with written descriptions of experimental design, sample attributes and methodology for studies of high-throughput gene expression and genomics. GEO is one of largest gene expression data repositories. As of September 2016, it contains microarray and RNA-Seq experiment results of 1 936 127 samples grouped into 73 415 datasets (named ‘data series’ in GEOs terminology). An up-to-date summary of GEO data types and content is provided at http://www.ncbi.nlm.nih.gov/geo/summary/.

According to GEO, a series record defines a group of related samples and provides a focal point and description of the whole study. Series records may also contain tables describing extracted data, summary conclusions or analyses. Each series record is assigned a unique GEO accession number (GSExxx) that summarizes an experiment. R package rentrez provides an R interface to the NCBI's EUtils API allowing users to search databases (20). We used rentrez to fetch a list of datasets that meets our query criteria (i.e. get Homo sapiens studies with sample size between 200 and 300, measured with GPL570 and provide CEL files).

#### Recount

Recount (19) is an online resource consisting of RNA-seq gene and exon counts from nearly 60 000 human RNA-seq samples from the sequence read archive (SRA). Recount is available in https://jhubiostatistics.shinyapps.io/recount/ and was last accessed on September 2016. It is a resource of processed and summarized expression data into concise gene count tables. In recount, data were processed using a single processing pipeline enabling thus a wider variety of downstream analyses. Recount R/Bioconductor package (https://github.com/leekgroup/recount) provides a convenient API for querying, downloading and analyzing the data. Each processed study consists of meta- and phenotype data, the expression levels of genes and their underlying exons and splice junctions and corresponding genomic annotation. We used recount as the main source of BioDataome’s RNA-Seq gene expression data.

### Gene expression and DNA methylation data sets

As of May 2017, we downloaded and processed gene expression data for Homo Sapiens and Mus Musculus samples from GEO and Homo Sapiens from recount. Table 1 shows a description of the different data set types currently hosted in BioDataome. In this paper, we show results only on the datasets with sample size greater than or equal to 40, as a snapshot of BioDataome. However, BioDataome is updated bi-weekly with ∼500 samples. Most of the publicly available data are microarrays, but most studies are now performed using RNA-seq. Affymetrix (now Thermo Fisher Scientific) Human Genome U133 Plus 2.0 Array is the most widely used gene expression array.

**Table 1.** Gene expression and DNA methylation data collections^a^

Species	Entity	Technology/ ID	Type	Resource
**Homo Sapiens (243 563)**	Gene expression	Affymetrix human genome U133 Plus 2.0 Array-GPL570 (*144639*) – 55%	*In situ* oligonucleotide	GEO
		Affymetrix human genome U133A Array-GPL96 (31 204)—12%		
		Affymetrix human gene 1.0 ST Array- GPL6244 (21 891) —8%		
	DNA methylation	Illumina human methylation450 BeadChip- GPL13534 (13 140)—5%	Oligonucleotide beads	
	Gene expression	Illumina HiSeq 2000- GPL11154 (32 689)—12%	Expression profiling by high throughput sequencing	Recount
**Mus Musculus (20 723)**	Gene expression	Affymetrix mouse genome 430 2.0 Array-GPL1261 (20 723) —8%	*In situ* oligonucleotide	GEO

a Biodataome includes 243 563 Homo Sapiens and 20 723 Mus Musculus samples. Gene expression data for Homo Sapiens result from three Affymetrix arrays (GPL570, GPL96 and GPL6244) and from RNA-Seq data from the recount (GPL11154). Gene expression data for Mus Musculus result from the most common array, GPL1261. DNA methylation result from GPL13534. Percentages next to sample counts represent the dataset percentage in BioDataome.

**Figure 1.**
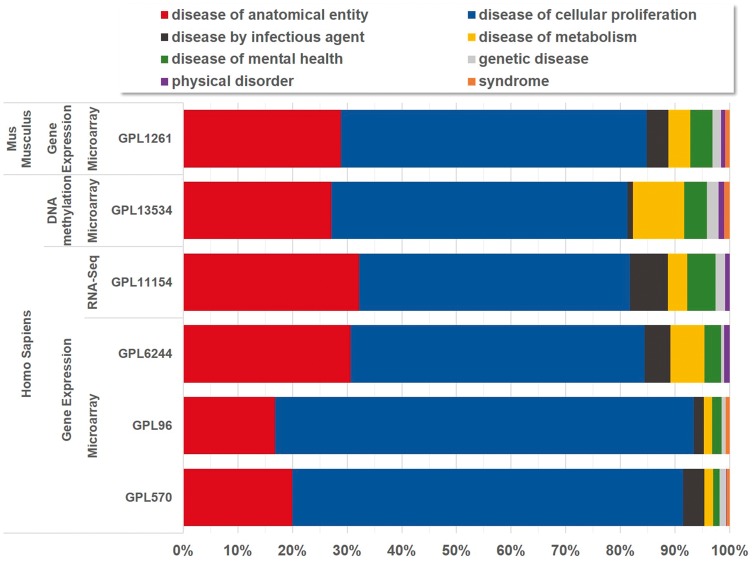
Disease distribution of BioDataome’s datasets per species and measured technology. Disease categories correspond to parent disease nodes according to D-O (http://disease-ontology.org/).

**Figure 2.**
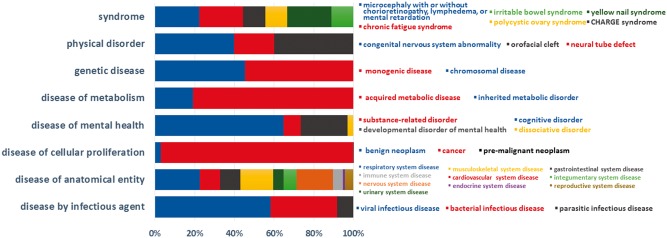
Dataset distribution per disease category on the children nodes of D-O.

Currently, BioDataome stores over 260 K samples from thousands of datasets, mostly Homo Sapiens gene expressions. All samples are commonly preprocessed based on each technology and automatically annotated with disease terms from the Disease-Ontology (D-O) (21). All disease terms can be traced back to their top-level parent node in the D-O. In Figure 1, we show the distribution of BioDataome datasets in the eight top level parent nodes of D-O on each technology and in Figure 2, we show how these top-level categories are distributed across their children nodes. Datasets related to cancer are the most prevalent in cellular proliferation diseases.

### Preprocessing of BioDatome data sets

#### Microarray gene expression preprocessing

Gene expression microarrays are the most widely used type of microarrays and have proved extremely useful in defining gene expression patterns for diagnosis, prognosis and prediction of clinical outcomes. Microarray gene expression data are high dimensional, noisy data. Several experimental technical or biological factors cause systematic variations in microarray data. Therefore, a crucial step in microarray data analysis is to evaluate the quality of the data and correct for potential technical or biological biases. This step is called preprocessing and ensures data comparability before any downstream analysis is performed. Typical preprocessing involves three steps: ‘background correction’, ‘normalization’ and ‘summarization’.

Normalization and summarization require multiple arrays to be analyzed simultaneously and thus most gene expression microarray preprocessing techniques are multi-array preprocessing techniques, using information from other samples to estimate probe level effects. This constitutes a prohibiting factor for their use in per sample preprocessing limiting thus their application in personalized medicine or in clinical settings where samples must be processed individually. To overcome such limitations, preprocessing techniques that handle individual samples have developed, two of the most popular being fRMA (9) and SCAN (10). One limitation of fRMA is that since it leverages large amounts of public data to construct a reference model upon which each feature on the microarray is compared and normalized, it requires an adequate number and diversity of reference samples for a given array to be deposited in public data repositories, like GEO, to be able to support external reference vectors for that array.

SCAN uses only data from a given array for normalization and thus does not require any array-specific ancillary samples. Moreover, SCAN can be used to process multiple arrays in parallel and thus speed-up the preprocessing time and also corrects for binding-affinity biases using only data from within a given sample. SCAN estimates the probe effect through a linear statistical model and distinguishes between background noise and biological signal with a mixture-modeling approach and adjusts for sample-level variations in expression intensity arising from array and batch effects. Probe-level values are standardized against the variance observed for other probes on the same array that have similar binding-affinity-adjusted intensities.

#### RNA-seq preprocessing

RNA sequencing (RNA-Seq) is an alternative technique to measure gene expression and has quickly become researchers’ preferred measuring technology for transcriptome analysis. Public sequencing data repositories such as the SRA, NIH's primary archive of high-throughput sequencing data, now hold >100 000 human RNA-Seq samples, and the size of the archive doubles approximately every 18 months (https://www.ncbi.nlm.nih.gov/sra/docs/sragrowth/). One difference of gene expression microarray data with RNA-Seq data is that in the later we are dealing with counts instead of just intensities, and for counts, most of the approaches that are currently used, are modeled with Poisson or negative binomial.

The majority of these archived samples are available only as raw sequencing reads. GEO stores summarized raw reads into gene counts, however, these expression level summarizations are heavily dependent on the processing pipelines, which can vary dramatically across the study. For a survey on best practices for RNA-Seq data analysis (22). We downloaded summarized count data as RangedSummarizedExperiment objects from Recount, on 233 datasets with sample size greater than or equal to 40 (accessed on December 2016). We followed the same preprocessing pipeline on all downloaded datasets and all the details and steps followed are described in BioDataome documentation (http://dataome.mensxmachina.org/documentation.html).

#### DNA methylation preprocessing

Epigenetics, among which DNA methylation (DNAm), one of several epigenetic mechanisms that cells use to control gene expression, are increasingly being studied for their role in disease development or to serve as biomarkers for patients at risk of developing a disease. In recent decades, researchers have learned a great deal about DNA methylation, including how it occurs and where, and they have also discovered that methylation is an important component in numerous cellular processes, including embryonic development, genomic imprinting, X-chromosome inactivation and preservation of chromosome stability. Given the many processes in which methylation plays a role, it is not surprising that variation in methylation are linked to several human diseases (23).

DNAm is interesting also because it’s inherited at mitosis and the enzymatic mechanism for mitotically copying methylation status is well understood (24).

The Illumina Infinium HumanMethylation450 BeadChip (450 K) is the most commonly used tool to assess genome-wide DNAm which measures 485 512 CpG sites, the majority of which are localized to regions that potentially could regulate gene expression and therefore are of possible clinical relevance (99% of the sites are localized to genes that have been well-characterized in RefSeq or sites outside of genes that are likely to regulate gene expression, such as promoter regions) (25). Minfi R/Bioconductor package (26), which is used to read, preprocess and analyze data from the Illumina 450k DNA methylation array, has utilities to convert the data into methylation measurements. We performed DNAm preprocessing to construct β values with the Illumina preprocessing algorithm and called the ‘mapToGenome’ method to associate the ∼450 000 loci of the HumanMethylation450 array with genomic locations.

## Automatic annotation

### Disease annotation

Medical document repositories, such as PubMed (https://www.ncbi.nlm.nih.gov/pubmed/) contain a huge amount of medical literature and are supported by NLM. Although automatic extraction of useful information from these online sources for their classification is challenging because these documents are unstructured and indexed by human experts, works on automating and improving biomedical document indexing (27) show promising results. Additionally, biomedical databases such as GEO hold a vast amount of sample measurements, however, the annotation of those measurements with phenotypic characteristics (i.e. sample's disease, tissue type and control/treatment) is not straightforward since they are encoded as free text. Furthermore, requirements for metadata annotation are minimal, so phenotypic information resides in multiple documents and physical locations. Such information may be included as text describing the experiment or protocol, sample and sampling descriptions, or may be found only in the published journal article that may accompany the submission.

Several attempts towards biomedical data annotation and specifically gene expression data have been performed to increase biomedical database utility for researchers. Most of them involve manual annotation by human curators, mainly domain experts (28). Automatic annotation of biomedical data in large public repositories is essential to enhance their reuse, given their vast amount of available data. ExpressionBlast (16) includes a text analysis system able to identify the various components of a dataset in GEO (i.e identify treatment vs. control samples, replicates and time series). CRowd Extracted Expression of Differential Signatures (CREEDS) (29) is a crowdsourcing project to annotate and reanalyze a large number of gene expression profiles from GEO. PubTator (30) is a web-based tool designed to assist manual biocuration and text annotation with advanced text-mining techniques. PubTator stores text-mined annotations for every article in PubMed.

**Figure 3.**
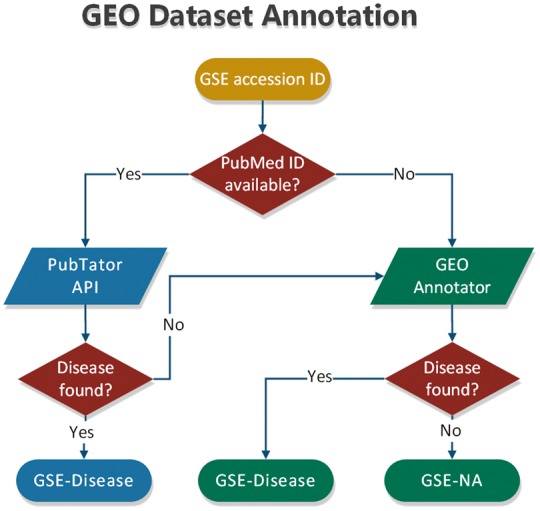
Flowchart of dataset annotation process.

To annotate datasets hosted in BioDataome, we programmatically retrieved text-mined results from PubTator through RESTful API. PubTator supports either PubMed ID queries or semantic search of several biological concepts (e.g. gene, disease and chemicals). This limits our ability to annotate datasets that are not accompanied by their related study. To extend our annotation capabilities, we developed a simple disease annotation tool for GEO studies, named GEO Annotator, i.e. based on GEO queries consisting of dataset accession ID and all disease terms from the D-O (21). In Figure 3, we show the flowchart of our annotation process that combines PubTator with GEO Annotator.

In most cases, PubTator returns a collection of disease terms for each dataset. The same holds for GEO Annotator. Therefore, we also developed a method that exploits the disease ontology to automatically select a minimum subset of disease terms to annotate each dataset. According to this method, we first map all disease terms of a collection to the disease ontology. If all the terms represent leaves of the disease ontology, we keep those that belong to the most common D-O subcategory. By D-O subcategory, we take the first children level in D-O (i.e. bacterial infectious disease, immune system disease, cancer, etc).

To validate our disease annotation methodology, we compared our automatic annotation results with the crowdsourced annotation of CREEDS (29). We downloaded the metadata of the manual disease signatures from CREED’s web portal and for the 169 overlapping GEO accession ids we compared the disease name in CREEDs with the respective name in BioDataome and consider a match when either a perfect string match is achieved or both disease terms are found in the title or abstract of the study. In Supplementary Table S1, we provide the results of this comparison along with a description and links that justify each match. Overall, in 82% (±6%) BioDatome’s disease annotation matches crowdsourced annotation as provided in CREEDS. In Supplementary Table S2, we have filtered out the mismatches and notice that in 18 out of the 30 mismatches BioDataome assigns a relative, however more general disease term.

### Discovering control samples

There are tens of thousands of studies in GEO covering different experimental designs (case-control, time-series, etc.); however, there is no straightforward way to determine it. Furthermore, it is not trivial to automatically discover treatment and control samples since a single study may involve multiple treatments and thus advanced text mining techniques should be applied to accurately annotate the samples of each individual study. In BioDataome, we automatically discover control samples from phenotype data in GEO by searching for predefined keywords that are often used to denote controls in specific columns of the phenotype matrix found in GEO. Although this approach is simple and only discovers control samples without providing any details on the treatment samples, it can be proved useful in cases where researchers are interested in merging control samples or even perform differential analysis in a simple two class experimental design.

To validate our results on automatic discovery of control samples, we again compared them with the crowdsourced annotation of CREEDS for the 169 overlapping studies. In total, we achieved an 84% (±2%) accuracy in automatically discovering control samples, as these are designated by the crowdsourced annotation in CREEDS. To help BioDataome users in automating their analysis tasks, along with the preprocessed datasets, we provide a metadata table for each dataset, that includes next to the sample names a column named ‘class’ in which we denote as control the samples that are classified as controls by our matching method and as unknown all other samples. We also include all phenotype information that we exploited to discover control samples, as downloaded from GEO, to support BioDataome users in their subsequent analysis.

## BioDataome overview and use

BioDataome is written in Laravel (version 5.2.0), a high-level PHP Web Framework, with a MySQL backend database. BioDataome includes advanced search functionality and provides the data in text format to allow universal use. The BioDataome landing page displays a search bar where users can type in any search term with autocomplete capabilities. On the search results, users can choose datasets of interest and download a list with relative information and download links. Users can narrow down the dataset list space by species, technology, disease or any combination of those. For example, a user can select all Homo Sapiens datasets related to Alzheimer’s disease that have no common samples in any other dataset.

### Data duplication

Several researchers reuse data, especially samples used as controls, and resubmit them to public repositories as new datasets. As genomic databases grow, the chance of inter and intra study duplication increases. Although this might not be an issue when analyzing individual experiments, it is a problem when performing meta-analyses. As the interest in making expression data reuse a routine part in genomic studies increases, careful consideration is necessary to address a number of challenges. If data reuse is not properly documented, it is misleading for the construction of secondary databases. Moreover, analysis of common samples can alter the identification of subsets of samples with clinical differences or the development of specific gene signatures. If mix-ups are present but undetected, the conclusions of the analysis might be affected and pollute the literature, as well as create a snowball effect for those who re-use the data. In BioDataome, users can query and filter such duplicates.

Given the potential for unrecognized duplication to falsely inflate prediction accuracy or other statistical analysis, duplicate sample detection should become a standard procedure for combining multiple genomic datasets. Waldron et al. (31) developed doppelgangR R/Bioconductor package to match duplicate cancer transcriptomes. They also discovered a sample mix-up in the TCGA Data (https://cancergenome.nih.gov/), which led to the removal of 50 profiles on August 25, 2015.

We identify common samples by comparing all pairwise combinations of the preprocessed datasets. For each dataset, we report all other datasets that share at least one common sample. We used Rfast R package (https://rfast.eu/), a collection of fast utility functions for data analysis, to speedup the comparisons.

**Figure 4.**
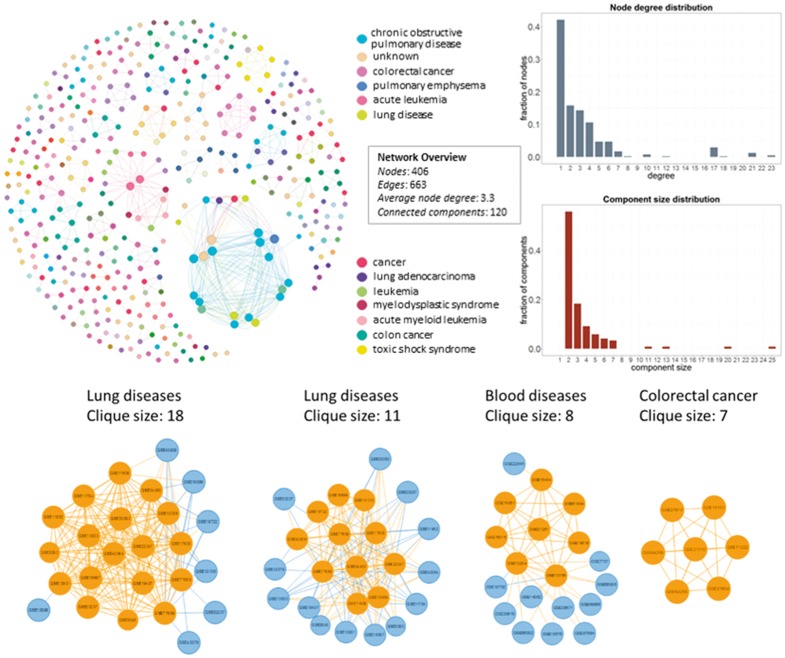
Network of inter-dataset duplicate samples (top left). Each node represents a dataset and edges connect datasets that share at least one sample. Node degree (top right) and component size (bottom right) distribution of the sample duplication network. The four largest maximal cliques (bottom). Orange represents clique nodes and blue the rest datasets of each component.

In Figure 4, we show the network datasets that share samples for the most widely used gene expression microarray, Affymetrix Human Genome U133 Plus 2.0 Array (GPL570). Each node represents a datasets and edges connect datasets that share at least one sample. The largest component in this network is constructed from datasets related to chronic obstructive pulmonary disease. All other datasets of this component are also related to lung diseases. Two of the largest maximal cliques are found in this component as shown in Figure 4 (bottom left). Although data reuse in GPL570 is high, relative to other technologies, most of the components in the data duplication graph, have the smallest node degree, indicating that data reuse could be easily extended.

**Figure 5.**
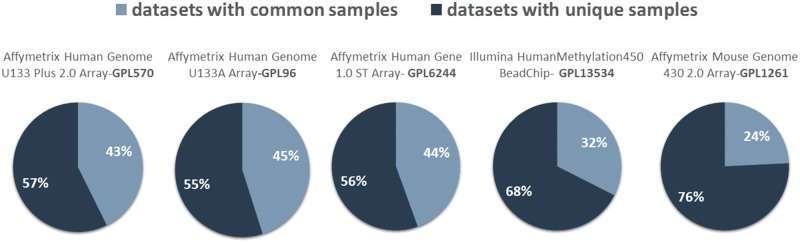
Datasets with (dark blue) and without (light blue) common samples for all arrays.

The extend of sample duplication in other human gene expression arrays is similar. In human DNA methylation array, a newer technology than gene expression, sample duplication is still low. We found no common samples in any of the RNA-Seq datasets. Not surprisingly, in mouse array, sample duplication is much lower compared to human arrays probably due to the highest cost effectiveness (Figure 5).

### Summary analytics at the gene level

#### Disease-wide analysis of gene expression

Large-scale microarray experiments can serve as a resource for a systematic understanding of global expression trends in the transcriptome under various experimental conditions. Current approaches have focused on identifying differentially expressed signatures from public gene expression repositories, mainly GEO (12) and ArrayExpress (13), and leverage them for the development of signature search engines (32, 16). Such approaches highly depend on the accurate annotation of the control and perturbation samples and other approaches like crowdsourcing have been developed recently to overcome these limitations (29). The major goal of microarray experiments so far has been the identification of differentially expressed genes, whose expression differs statistically significantly in a case-control experimental design, with the overall aim to discover biomarkers for diagnosis and prognosis. So far, researchers have been focused on developing sophisticated statistical methods to achieve more reliable identification of differentially expressed genes, either from microarray (33) or from RNA-Seq (34) experiments.

Despite the enormous impact that differential gene expression studies have in unraveling the molecular processes underlying disease pathophysiology, several shortcomings such as the use of different preprocessing or statistical methods, genetic or epigenetic variation, inherent stochasticity of biological processes and the heterogeneity of complex diseases, have made the results of those studies often inconsistent and not easily reproduced. Recently, Menche et al. (35) showed that genes that are differentially expressed between cases and controls are not up-regulated or down-regulated in each individual with the phenotype and propose the construction of individual perturbation expression profiles for a given sample as a ‘barcode,’ representing the genes that are up-regulated or down-regulated compared to the control group. Concurrently, as biology has now adapted a holistic approach to decipher the complexity of biological systems, approaches of networks and systems biology promise to shed light into the complicated mechanism of disease pathophysiology. For example, Zickenrott et al. (36) proposed a method for predicting disease–gene–drug relationships based on the reconstruction of phenotype-specific gene regulatory networks underlying phenotypic differences between disease and healthy states, solely relying on differential gene expression data.

With BioDataome, it has become possible to analyze the global expression trends of a gene in diverse biological samples under various diseases. Since a common fluorescence intensity normalization has been applied to all gene expression microarray data, researchers can easily query and gather all relevant datasets to their studies and directly apply their methods, from a personalization to a systems level, without wasting valuable time trying to discover interesting datasets. Here, we demonstrate BioDataome as a gene-disease exploratory tool, through the creation of a disease-wide expression profile for individual genes, which provides an estimation of the gene-specific distribution of expression levels under various diseases.

To compare the shapes of distributions, we performed two statistical tests, the first measuring skewness as a measure of symmetry, and the second kurtosis as a measure of whether the data are heavy-tailed or light-tailed relative to a normal distribution (37).

We calculated the combined *P*-value of the two statistical tests as $p=\max\left\{p_{s},p_{k}\right\}\;$for each gene (38).

First, we queried BioDataome for all disease annotated datasets of sample size >40 of the most popular array (GPL570) and computed the false discovery rate (FDR) adjusted *P*-values for all probes and diseases to account for multiple comparisons. Then, we mapped probes to genes using the R/Bioconductor annotation hgu133plus2.db (http://bioconductor.org/packages/hgu133plus2.db/) and calculated *P*-values for each gene as the median *P*-values of probes measuring the same gene.

**Figure 6.**
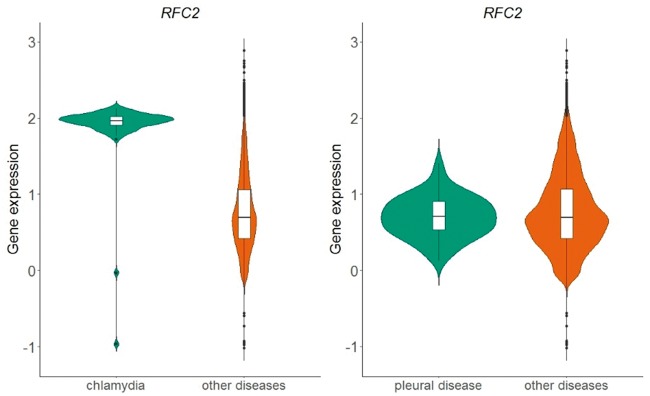
Violin plots of *RFC2* gene expression in chlamydia samples vs. all other samples in GPL570 array (left plot) and in pleural disease samples vs. all other samples in GPL570 array (right plot). P-value in the chlamydia case is almost zero, meaning that the distributions (green vs. orange) differ statistically significantly, whereas in the pleural disease case, the combined p-value of the two statistical tests (skewness, kurtosis) was 0.82 and thus the null hypothesis that the shapes of the two distributions are similar could not be rejected.

The above process resulted in a table of 166 different diseases and 20 534 genes. Each cell in this table represents whether the distribution of normalized expression values of a specific gene is statistically significantly different than the respective distribution of all other diseases. In Figure 6, we show an example of the replication factor C subunit 2 (*RFC2*) gene, located in chromosome 7, which encodes a member of the activator 1 small subunits family. The distribution of *RFC2* expression in datasets related to chlamydia disease differs significantly from the distribution of all other datasets of the same array (Figure 6-left). This is not true for pleural disease, where no statistical significant difference was detected for the same gene (Figure 6-right).

**Figure 7.**
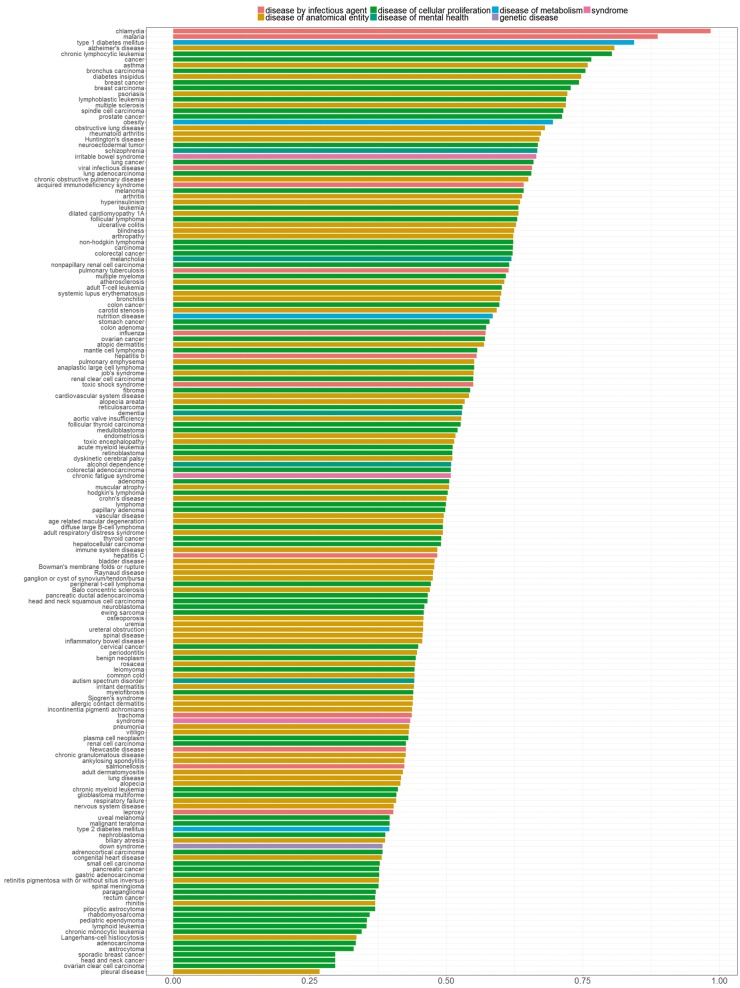
Percentage of genes with statistically significantly different distribution among diseases. Colors indicate disease categories according to the D-O.

In Figure 7, we sorted diseases based on the percentage of genes with distinctive distributions. We refer to genes showing a distinctive expression distribution for a specific disease as ‘disease-specific genes’. Bar colors correspond to the different parent nodes of diseases according to the D-O. In the first five diseases, 80% or more of all genes were identified as disease specific genes, whereas in the last four diseases <30% of all genes are disease specific.

#### Gene set analysis

To retrieve a functional profile for a specific gene set resulted from high-throughput experimental techniques such as microarray, a common way is to leverage the biological knowledge, such as Gene Ontology (GO) (http://www.geneontology.org/) and Kyoto Encyclopedia of Genes and Genomes (KEGG) (http://www.genome.jp/kegg/), for identifying predominant biological themes of a collection of genes. This is useful for finding out if the specific gene set of interest (i.e. a list of differentially expressed genes from a case-control study) are associated with a certain biological process or molecular function.

**Figure 8.**
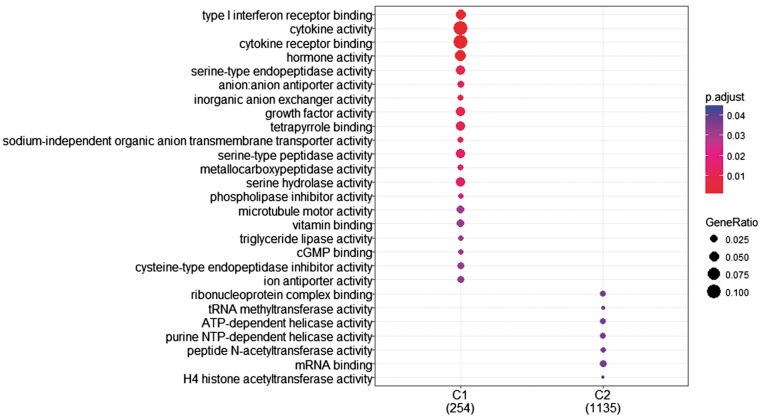
GO enrichment analysis of the two (C1: genes with statistically significantly different distributions in all diseases and C2: genes that are statistically significantly different distributions in at most 20 diseases). GO annotation was based on Homo Sapiens OrgDb object. Color gradient ranges from red to blue. Red indicates low adjusted p-values (high enrichment), and blue indicates high adjusted p-values (low enrichment). Dot size corresponds to the count of ‘GeneRatio’.

The research question that arises at this point is whether we can identify genes with differentiated expression distribution in many diseases and genes that are differentiated in one or few diseases and how these gene sets are functionally compared. To investigate this, we proceeded as follows. First, we created two gene sets of interest to functionally analyze them. The first includes all genes with differentiated expression distributions in all diseases. These are 366 genes in total (G1). The second includes genes with differentiated expression distributions in at most 20 diseases. These are 1285 genes (G2). Then we used the R-package, ‘clusterProfiler’ (39), to identify clusters enriched in GO-functions. ‘compareCluster’ with the function parameter set to ‘enrichGO’ lists molecular function in clusters if they are found to be significantly enriched. P-value cutoff was set to its default value (0.05), along with all other parameters. As shown in Figure 8, a subset of G1, noted as C1 and a subset of G2, noted as C2 were found to enrich mutually exclusive molecular functions. Cytokine activity and cytokine receptor binding are highly enrichment for most of the genes in C1.

**Figure 9.**
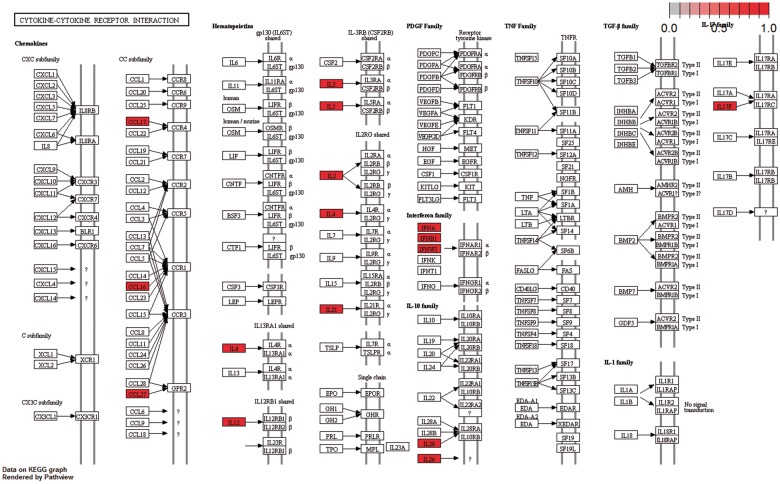
Cytokine–cytokine receptor interaction pathway: KEGG pathway with the highest enrichment score on the gene set of differentiated genes in all diseases. Highlighted with red are the genes that belong to this gene set. Pathway was visualized with pahtview R/Bioconductor package (40).

Moreover, the cytokine–cytokine receptor interaction pathway had the highest enrichment score in KEGG Gene Set Enrichment Analysis. *P*-values were based on 1000 permutations and subsequently adjusted for multiple testing using the Benjamini–Hochberg procedure to control the FDR (Figure 9).

## Conclusions

By maximizing the use of existing data and the reusability of newly generated omics data, important new discoveries will be made in a cost-effective way. When relevant data sets are combined in a largescale analysis, new biological insights appear that would be impossible to obtain from the individual studies. BioDataome is a database of uniformly preprocessed omics datasets. To the best of our knowledge, there is still no single place where researchers can download thousands of ready for downstream analysis omics datasets, which have been annotated with D-O terms and at the same time provide information on sample duplication and whether a sample can be considered as a control. For example, a user can select all Homo Sapiens gene expression datasets related to Alzheimer’s disease that have no common samples in any other dataset. This combination makes BioDataome a competitive tool which promotes and accelerates research work on integrative and large-scale genomic studies. We plan to continually maintain and expand the BioDataome while keeping it free and open resource.

## Supplementary data

Supplementary data are available at *Database* Online

## Funding

This project has received funding from the European Research Council (ERC) under the European Union’s Seventh Framework Programme (FP/2007-2013) (grant agreement no 617393).

*Conflict of interest*. None declared.

## Supplementary Material

Supplementary Table 1

Supplementary Table 2
