# Supplementary material for: BioDataome: a collection of uniformly preprocessed and automatically annotated datasets for data-driven biology
Source: Database (Oxford). 2018 Mar 2;2018:bay011. doi: 10.1093/database/bay011 (PMC5836265; doi:10.1093/database/bay011)
Supplement: Supplementary Table 1 [file bay011_supp_table1.pdf]

Table 1: Disease annotation as provided by CREEDS and BioDataome. Match equals to 1 when the two annotations agree. Comments and links justify a match when both terms are correct. Overall, in 82% (±6%) BioDatome's disease annotation matches crowdsourced annotation as provided in CREEDS.

GSE	creeds	biodataome	Matches	Comments	Link
GSE10325	systemic lupus erythematosus	systemic lupus erythematosus	1		
GSE10575	arthropathy	osteoarthritis	1	Migratory chondrogenic progenitor cells from repair tissue during the later stages of human osteoarthritis	https://www.ncbi.nlm.nih.gov/geo/query/acc.cgi?acc=GSE10575
GSE10631	LGLL - Large granular lymphocytic leukemia	leukemia			
GSE10946	polycystic ovary syndrome	polycystic ovary syndrome	1		
GSE10971	serous cystadenocarcinoma	ovarian cancer	1	Serous cystadenocarcinoma is a type of tumor in the cystadenocarcinoma grouping. Most commonly the primary site of serous cystadenocarcinoma is the ovary.	https://en.wikipedia.org/wiki/Serous_cystadenocarcinoma
GSE11348	Rhinovirus infection	viral infectious disease	1	The rhinovirus is the most common viral infectious agent in humans	https://en.wikipedia.org/wiki/Rhinovirus
GSE11524	sickle cell anemia	sickle cell anemia	1		
GSE11886	ankylosing spondylitis	ankylosing spondylitis	1		
GSE12452	nasopharynx carcinoma	spindle cell carcinoma			
GSE12649	Schizophrenia	bipolar disorder	1	Accumulating evidence suggests that mitochondrial dysfunction underlies the pathophysiology of bipolar disorder (BD) and schizophrenia (SZ)	https://www.ncbi.nlm.nih.gov/geo/query/acc.cgi?acc=GSE12649
GSE12679	bipolar disorder	schizophrenia	1	Brain tissue from patients with schizophrenia and bipolar disorder and healthy controls	https://www.ncbi.nlm.nih.gov/geo/query/acc.cgi?acc=GSE12679
GSE13355	Psoriasis vulgaris	psoriasis	1	Psoriasis vulgaris, makes up about 90 percent of psoriasis cases	https://en.wikipedia.org/wiki/Psoriasis
GSE13887	systemic lupus erythematosus	systemic lupus erythematosus	1		
GSE14407	ovarian cancer	ovarian cancer	1		
GSE14905	psoriasis	psoriasis	1		
GSE15090	facioscapulohumeral muscular dystrophy	facioscapulohumeral muscular dystrophy	1		
GSE15471	pancreatic ductal adenocarcinoma	pancreatic ductal adenocarcinoma	1		

GSE15824	oligodendroglioma	glioblastoma multiforme	1	Glioblastoma multiforme is a malignant glioma and the most aggressive and most common primary brain tumor. Oligodendroglioma is a type of glioma	
GSE16032	asthma	asthma	1		
GSE16461	relapsing-remitting multiple sclerosis	rheumatoid arthritis			
GSE16464	osteoarthritis	osteoarthritis	1		
GSE16515	pancreatic cancer	pancreatic cancer	1		
GSE16538	pulmonary sarcoidosis	pulmonary sarcoidosis	1		
GSE16715	Williams-Beuren syndrome	williams-beuren syndrome	1		
GSE16728	sickle cell anemia	sickle cell anemia	1		
GSE17612	schizophrenia	schizophrenia	1		
GSE18632	amyotrophic lateral sclerosis type 10	frontotemporal dementia	1	Inclusions of TDP-43 are hallmarks of frontotemporal dementias and amyotrophic lateral sclerosis	https://www.ncbi.nlm.nih.gov/geo/query/acc.cgi?acc=GSE18632
GSE18670	pancreatic cancer	pancreatic ductal adenocarcinoma	1	Pancreatic ductal adenocarcinoma (PDAC) is the most common malignancy of the pancreas.	https://www.pancreapedia.org/reviews/pancreatic-ductal-adenocarcinoma
GSE19650	pancreatic invasive intraductal papillary-mucinous carcinoma	pancreatic cancer	1	Intraductal papillary-mucinous neoplasm (IPMN) of the pancreas is a precursor lesion of pancreatic cancer.	https://www.ncbi.nlm.nih.gov/geo/query/acc.cgi?acc=GSE19650
GSE20948	hepatitis C	spindle cell carcinoma			
GSE21369	idiopathic interstitial pneumonia	idiopathic pulmonary fibrosis	1	idiopathic pulmonary fibrosis is a subtype of idiopathic interstitial pneumonia	https://www.ncbi.nlm.nih.gov/geo/query/acc.cgi?acc=GSE21369
GSE21422	invasive ductal carcinoma	ductal carcinoma in situ	1		
GSE21942	multiple sclerosis	multiple sclerosis	1		
GSE22619	ulcerative colitis	ulcerative colitis	1		
GSE23952	pancreatic cancer	pancreatic cancer	1		
GSE24206	idiopathic pulmonary fibrosis	idiopathic pulmonary fibrosis	1		
GSE24514	colorectal adenocarcinoma	spindle cell carcinoma			
GSE26299	glaucoma	glaucoma	1		
GSE26910	breast cancer	cancer			
GSE26969	intracranial aneurysm	Bowman's membrane folds or			
GSE27011	asthma	asthma	1		
GSE28619	alcoholic hepatitis	alcoholic hepatitis	1		

GSE29639	precursor B lymphoblastic lymphoma/leukemia	leukemia	1		
GSE30153	lupus erythematosus	systemic lupus erythematosus	1	Systemic lupus erythematosus (SLE), also known simply as lupus	https://en.wikipedia.org/wiki/Systemic_lupus_erythematosus
GSE3167	Urothelial carcinoma in situ	transitional cell carcinoma	1	In this study, we used microarray expression profiling to examine the gene expression patterns in superficial transitional cell carcinoma	https://www.ncbi.nlm.nih.gov/geo/query/acc.cgi?acc=GSE3167
GSE32323	colorectal cancer	colorectal cancer	1		
GSE32924	atopic dermatitis	atopic dermatitis	1		
GSE3325	prostate cancer	prostate cancer	1		
GSE34299	colon cancer	melanoma			
GSE34308	adrenoleukodystrophy	teratoma			
GSE34526	polycystic ovary syndrome	polycystic ovary syndrome	1		
GSE36398	facioscapulohumeral muscular dystrophy	facioscapulohumeral muscular dystrophy	1		
GSE36474	multiple myeloma	multiple myeloma	1		
GSE36700	systemic lupus erythematosus	synovitis	1	Gene expression profiles in synovial biopsies from patients with arthritis	https://www.ncbi.nlm.nih.gov/geo/query/acc.cgi?acc=GSE36700
GSE36980	Alzheimer's disease	vascular dementia	1	Three-way ANOVA of microarray data from frontal cortex, temporal cortex and hippocampus with presence/absence of AD and vascular dementia	https://www.ncbi.nlm.nih.gov/geo/query/acc.cgi?acc=GSE36980
GSE3744	breast cancer	cancer			
GSE38010	multiple sclerosis	multiple sclerosis	1		
GSE38680	Pompe disease, infantile-onset form	glycogen storage disease II	1	Glycogen storage disease type 2, also known as Pompe disease	https://rarediseases.info.nih.gov/diseases/5714/glycogen-storage-disease-type-2
GSE39452	prostate cancer	prostate cancer	1		
GSE39621	Niemann-Pick disease	gaucher's disease	1	Niemann-Pick disease type A or Gaucher disease	https://rarediseases.org/rare-diseases/niemann-pick-disease-type-c/
GSE4036	Schizophrenia	fetal alcohol syndrome	1	Studying the basis of learning disabilities and of diseases that show deficits in PPI such as fetal alcohol syndrome and schizophrenia	https://www.ncbi.nlm.nih.gov/geo/query/acc.cgi?acc=GSE4036

GSE4107	colon cancer	familial adenomatous polyposis	1	Familial adenomatous polyposis (FAP) is an inherited disorder characterized by cancer of the large intestine (colon) and rectum	https://ghr.nlm.nih.gov/condition/familial-adenomatous-polyposis
GSE4183	colon adenoma	ulcerative colitis	1	Functional classification of precancerous adenoma, different stage colorectal carcinomas (CRC) and inflammatory bowel diseases (IBD)	https://www.ncbi.nlm.nih.gov/geo/query/acc.cgi?acc=GSE4183
GSE4250	Hereditary gingival fibromatosis	gingival fibromatosis	1		
GSE4290	oligodendroglioma	neuroectodermal tumor	1	Both are types of glioma brain tumour	http://www.cancerresearchuk.org/about-cancer/brain-tumours/types
GSE4302	asthma	asthma	1		
GSE43292	atherosclerosis	atherosclerosis	1		
GSE44723	idiopathic pulmonary fibrosis	idiopathic pulmonary fibrosis	1		
GSE47552	multiple myeloma	multiple myeloma	1		
GSE4757	Alzheimer's disease	alzheimer's disease	1		
GSE48060	acute myocardial infarction	myocardial infarction	1		
GSE49036	Lewy body dementia	parkinson's disease	1	Subjects with incidental Lewy body disease (iLBD) may represent the premotor stage of Parkinson’s disease (PD)	https://www.ncbi.nlm.nih.gov/geo/query/acc.cgi?acc=GSE49036
GSE49515	stomach cancer	hepatocellular carcinoma	1	pancreatic carcinoma, gastric carcinoma and hepatocellular carcinoma (HCC) were identified using Affymetrix gene arrays	https://www.ncbi.nlm.nih.gov/geo/query/acc.cgi?acc=GSE49515
GSE5109	obesity	obesity	1		
GSE5388	bipolar disorder	bipolar disorder	1		
GSE5392	bipolar disorder	bipolar disorder	1		
GSE54958	papillary thyroid carcinoma	papillary adenoma			
GSE54992	tuberculosis	tuberculosis	1		
GSE5563	Vulvar intraepithelial tumor	cancer			
GSE5764	invasive ductal carcinoma	breast cancer	1	We examined ten mastectomy specimens from postmenopausal breast cancer patients	https://www.ncbi.nlm.nih.gov/geo/query/acc.cgi?acc=GSE5764
GSE58208	hepatocellular carcinoma	spindle cell carcinoma			
GSE58435	Turner syndrome	turner syndrome	1		

GSE6054	familial hypercholesterolemia	familial hypercholesterolemia	1		
GSE6088	familial hypercholesterolemia	familial hypercholesterolemia	1		
GSE6281	allergic contact dermatitis	allergic contact dermatitis	1		
GSE6364	endometriosis	infertility			
GSE63941	esophagus squamous cell carcinoma	cancer			
GSE65144	anaplastic thyroid carcinoma	spindle cell carcinoma			
GSE6575	autistic disorder	autism spectrum disorder	1		
GSE6613	Parkinson's disease	Brown's tendon sheath			
GSE6691	chronic lymphocytic leukemia	macroglobulinemia	1	Gene expression profiling of B lymphocytes and plasma cells from WaldenstrÖm's macroglobulinemia	https://www.ncbi.nlm.nih.gov/geo/query/acc.cgi?acc=GSE6691
GSE6740	Human immunodeficiency virus infectious disease	acquired immunodeficiency syndrome	1	data from frontal cortex, temporal cortex and hippocampus with presence/absence of AD and vascular dementia	http://www.stanfordchildrens.org/en/topic/default?id=acquired-immune-deficiency-syndrome-aidshuman-immunodeficiency-virus-hiv-90-P02427
GSE6764	hepatocellular carcinoma	cancer			
GSE6798	polycystic ovary syndrome	polycystic ovary syndrome	1		
GSE6872	Teratospermia	infertility	1	We report here the spermatozoal transcript profiles characteristic of normally fertile individuals and infertile males suffering from a consistent and severe teratozoospermia	https://www.ncbi.nlm.nih.gov/geo/query/acc.cgi?acc=GSE6872
GSE7305	endometriosis	infertility			
GSE7486	epilepsy syndrome	epilepsy	1		
GSE7621	Parkinson's disease	alcohol dependence			
GSE7753	JRA - Juvenile rheumatoid arthritis	arthropathy	1	Juvenile idiopathic arthritis, is the most common type of arthritis in children	https://www.mayoclinic.org/diseases-conditions/juvenile-rheumatoid-arthritis/basics/definition/con-20014378
GSE8157	polycystic ovary syndrome	polycystic ovary syndrome	1		
GSE8586	bronchopulmonary dysplasia	lung disease	1	Bronchopulmonary dysplasia is a chronic lung disease	https://en.wikipedia.org/wiki/Bronchopulmonary_dysplasia
GSE8762	Huntington's disease	huntington's disease	1		

GSE8835	chronic lymphocytic leukemia	chronic lymphocytic leukemia	1		
GSE9452	ulcerative colitis	ulcerative colitis	1		
GSE9476	acute myeloid leukemia	acute myeloid leukemia	1		
GSE9692	Septic Shock	toxic shock syndrome	1	TSS is a special form of septic shock caused by the toxins of the *Staphylococcus* and *Streptococcus* bacteria	http://www.medbroadcast.com/condition/getcondition/toxic-shock-syndrome
GSE9750	cervical cancer	cervical cancer	1		
GSE9877	sickle cell anemia	anemia	1		
GSE10162	Nephrolithiasis	dent disease	1	The fingerprint of these gene changes may help us to understand the phenotype of Dent disease	https://www.ncbi.nlm.nih.gov/geo/query/acc.cgi?acc=GSE10162
GSE10167	Treacher Collins syndrome	treacher collins syndrome	1		
GSE11343	diabetic neuropathy	diabetic	1		
GSE11494	Streptococcal tonsillitis	tonsillitis;tonsillitis	1		
GSE11686	cerebral palsy	duchenne muscular dystrophy			
GSE11686	cerebral palsy	duchenne muscular dystrophy			
GSE11971	childhood type dermatomyositis	adult dermatomyositis;childhood type	1		
GSE13083	Barrett's esophagus	barrett's esophagus;barrett's esophagus	1		
GSE13597	nasopharynx carcinoma	ataxia telangiectasia			
GSE1420	esophagus adenocarcinoma	barrett's esophagus	1	Barrett's esophagus, Barrett's-associated adenocarcinomas and normal esophageal epithelium	https://www.ncbi.nlm.nih.gov/geo/query/acc.cgi?acc=GSE1420
GSE14277	lung cancer	lung cancer;adenocarcinoma	1		
GSE1462	MELAS syndrome	mitochondrial encephalomyopathy;ophthalmoplegia	1	Extremely variable clinic and genetic features characterize Mitochondrial Encephalomyopathy Disorders	https://www.ncbi.nlm.nih.gov/geo/query/acc.cgi?acc=GSE1462
GSE15568	cystic fibrosis	cystic fibrosis	1		
GSE1650	chronic obstructive pulmonary disease	chronic obstructive pulmonary disease	1		
GSE1786	Senescence	chronic obstructive pulmonary			
GSE18064	Rift Valley fever	rift valley	1		
GSE18965	asthma	asthma	1		
GSE19286	atherosclerosis	atherosclerosis	1		
GSE19780	Huntington's disease	huntington's disease	1		
GSE20602	nephrosclerosis	nephrosclerosis	1		

GSE20844	glomerulosclerosis	glomerulosclerosis	1		
GSE23832	multiple sclerosis	multiple sclerosis	1		
GSE24250	Huntington's disease	neurodegenerative disease			
GSE25673	schizophrenia	schizophrenia	1		
GSE26001	Huntington's disease	huntington's disease	1		
GSE27131	swine influenza	influenza	1		
GSE27628	psoriasis	psoriasis	1		
GSE28315	epidermolysis bullosa simplex	epidermolysis bullosa simplex	1		
GSE3100	cystic fibrosis	cystic fibrosis	1		
GSE31106	colorectal adenocarcinoma	colorectal cancer	1		
GSE34619	Barrett's esophagus	adenocarcinoma			
GSE34925	breast cancer	breast cancer	1		
GSE3524	Squamous cell carcinoma of mouth	oral squamous cell carcinoma	1		
GSE3554	glaucoma	glaucoma	1		
GSE35561	Down syndrome	microcephaly with or without chorioretinopathy, lymphedema, or mental			
GSE3583	Huntington's disease	huntington's disease	1		
GSE3585	dilated cardiomyopathy	dilated cardiomyopathy	1		
GSE3889	Hypercholesteremia	cholestasis			
GSE41649	allergic asthma	allergic asthma	1		
GSE42589	cleft lip	cancer	1	Previous findings have appointed an aetiological overlap between NSCL/P and cancer, and alterations in similar biological pathways may underpin both conditions.	https://www.ncbi.nlm.nih.gov/geo/query/acc.cgi?acc=GSE42589
GSE44025	leukemia	leukemia	1		
GSE4646	Meningococcal infection	meningitis	1	Signaling of Neisseria meningitidis MC58 mutants to primary human umbilical vein endothelial cells (HUVEC)	
GSE474	obesity	obesity	1		

GSE48280	dermatomyositis	inclusion body myositis	1	RNA from MHC-I-positive myofibers were obtained from muscle biopsies of 5 patients with dermatomyositis, 5 with polymyositis, 4 with inclusion body myositis and normal looking fibers from healthy controls.	https://www.ncbi.nlm.nih.gov/geo/query/acc.cgi?acc=GSE48280
GSE48301	polycystic ovary syndrome	polycystic ovary syndrome	1		
GSE48964	morbid obesity	atherosclerosis	1	The adipose tissue is an endocrine regulator and a risk factor for atherosclerosis	https://www.ncbi.nlm.nih.gov/geo/query/acc.cgi?acc=GSE48964
GSE5090	polycystic ovary syndrome	polycystic ovary syndrome	1		
GSE5389	bipolar disorder	bipolar disorder	1		
GSE5390	Down syndrome	microcephaly with or without chorioretinopathy, lymphedema, or mental			
GSE57178	urticaria	urticaria	1		
GSE5788	Leukemia, Chronic T-Cell	lymphoma	1	T-cell prolymphocytic leukemina (T-PLL) is an agressive lymphoma derived from mature T-cells	https://www.ncbi.nlm.nih.gov/geo/query/acc.cgi?acc=GSE5788
GSE6011	Duchenne muscular dystrophy	duchenne muscular dystrophy	1		
GSE60502	hepatocellular carcinoma	hepatocellular carcinoma	1		
GSE62632	autism spectrum disorder	rett syndrome			
GSE6399	Emery-Dreifuss muscular dystrophy	emery-dreifuss muscular dystrophy	1		
GSE6461	synovial sarcoma	synovial sarcoma	1		
GSE64756	in situ carcinoma	carcinoma	1		
GSE6710	Psoriasis vulgaris	psoriasis	1		
GSE6980	multiple myeloma	multiple myeloma	1		
GSE8000	Emery-Dreifuss muscular dystrophy	cardiomyopathy	1	Activation of MAPK pathways links LMNA mutations to cardiomyopathy in Emery-Dreifuss muscular dystrophy	https://www.ncbi.nlm.nih.gov/geo/query/acc.cgi?acc=GSE8000
GSE9574	breast cancer	breast cancer	1		

Total matches	139
%percentage	82%
