# Supplementary material for: BioDataome: a collection of uniformly preprocessed and automatically annotated datasets for data-driven biology
Source: Database (Oxford). 2018 Mar 2;2018:bay011. doi: 10.1093/database/bay011 (PMC5836265; doi:10.1093/database/bay011)
Supplement: Supplementary Table 2 [file bay011_supp_table2.pdf]

Table 2: All mismatches of CREEDS and BioDataome comparison. In 18 out of 30 mismatches BioDataome assigns a relative, however more general disease term.

GSE	creeds	biodataome
GSE10631	LGLL - Large granular lymphocytic leukemia	leukemia
GSE12452	nasopharynx carcinoma	spindle cell carcinoma
GSE16461	relapsing-remitting multiple sclerosis	rheumatoid arthritis
GSE20948	hepatitis C	spindle cell carcinoma
GSE24514	colorectal adenocarcinoma	spindle cell carcinoma
GSE26910	breast cancer	cancer
GSE26969	intracranial aneurysm	Bowman's membrane folds or rupture
GSE34299	colon cancer	melanoma
GSE34308	adrenoleukodystrophy	teratoma
GSE3744	breast cancer	cancer
GSE54958	papillary thyroid carcinoma	papillary adenoma
GSE5563	Vulvar intraepithelial tumor	cancer
GSE58208	hepatocellular carcinoma	spindle cell carcinoma
GSE6364	endometriosis	infertility
GSE63941	esophagus squamous cell carcinoma	cancer
GSE65144	anaplastic thyroid carcinoma	spindle cell carcinoma
GSE6613	Parkinson's disease	Brown's tendon sheath syndrome
GSE6764	hepatocellular carcinoma	cancer
GSE7305	endometriosis	infertility
GSE7621	Parkinson's disease	alcohol dependence
GSE11686	cerebral palsy	duchenne muscular dystrophy
GSE11686	cerebral palsy	duchenne muscular dystrophy
GSE13597	nasopharynx carcinoma	ataxia telangiectasia
GSE1786	Senescence	chronic obstructive pulmonary disease
GSE24250	Huntington's disease	neurodegenerative disease
GSE34619	Barrett's esophagus	adenocarcinoma
GSE35561	Down syndrome	microcephaly with or without chorioretinopathy, lymphedema, or mental retardation
GSE3889	Hypercholesteremia	cholestasis
GSE5390	Down syndrome	microcephaly with or without chorioretinopathy, lymphedema, or mental retardation
GSE62632	autism spectrum disorder	rett syndrome
